# Functionalisation of Detonation Nanodiamond for Monodispersed, Soluble DNA-Nanodiamond Conjugates Using Mixed Silane Bead-Assisted Sonication Disintegration

**DOI:** 10.1038/s41598-017-18601-6

**Published:** 2018-01-15

**Authors:** Robert Edgington, Katelyn M. Spillane, George Papageorgiou, William Wray, Hitoshi Ishiwata, Mariana Labarca, Sergio Leal-Ortiz, Gordon Reid, Martin Webb, John Foord, Nicholas Melosh, Andreas T. Schaefer

**Affiliations:** 1 The Francis Crick Institute, 1 Midland Rd, Kings Cross, London, NW1 1AT UK; 2 Department of Materials Science and Engineering, Stanford University, Stanford, California 94305 United States; 3 Department of Chemistry, University of Oxford, Oxford, OX1 3TA UK; 4 Department of Neuroscience, Physiology & Pharmacology, University College London, London, UK; 5 Present Address: Department of Physics, King’s College London, London, WC2R 5 2LS, United Kingdom

## Abstract

Nanodiamonds have many attractive properties that make them suitable for a range of biological applications, but their practical use has been limited because nanodiamond conjugates tend to aggregate in solution during or after functionalisation. Here we demonstrate the production of DNA-detonation nanodiamond (DNA-DND) conjugates with high dispersion and solubility using an ultrasonic, mixed-silanization chemistry protocol based on the *in situ* Bead-Assisted Sonication Disintegration (BASD) silanization method. We use two silanes to achieve these properties: (1) 3-(trihydroxysilyl)propyl methylphosphonate (THPMP); a negatively charged silane that imparts high zeta potential and solubility in solution; and (2) (3-aminopropyl)triethoxysilane (APTES); a commonly used functional silane that contributes an amino group for subsequent bioconjugation. We target these amino groups for covalent conjugation to thiolated, single-stranded DNA oligomers using the heterobifunctional crosslinker sulfosuccinimidyl 4-(*N*-maleimidomethyl)cyclohexane-1-carboxylate (Sulfo-SMCC). The resulting DNA-DND conjugates are the smallest reported to date, as determined by Dynamic Light Scattering (DLS) and Atomic Force Microscopy (AFM). The functionalisation method we describe is versatile and can be used to produce a wide variety of soluble DND-biomolecule conjugates.

## Introduction

Nanodiamonds (NDs) are an attractive nanoparticle vehicle for biological applications due to their high chemical stability, low cytotoxicity, and their unique optical properties^1,2^. Their bulk composition of strong sp^3^ and sp^2^ carbon bonds render chemical stability and inertness in physiological environments and high cellular biocompatibility for negatively charged NDs^3–6^. Their derivative, fluorescent NDs, contain impurities such as nitrogen and silicon vacancies that emit bright and stable fluorescence^3,7–11^. Fluorescent NDs thus have a diverse range of potential applications in the life sciences including imaging, cell tracking, labeling, tissue scaffolding, diagnosis, and drug delivery^2,7,12–18^.

For NDs to be useful for biological applications they must remain monodispersed and soluble in solution, and have functionalisable surfaces for subsequent grafting of biomolecules. Monodispersity and solubility require that the ND surface is sufficiently charged and homogeneous so that counterions uniformly accumulate around the periphery and drive isotropic repulsive forces between neighbouring NDs. Functionalising an ND often decreases its solvency, however, by introducing non-polar compounds (e.g., aromatic groups and/or hydrocarbons) and increasing the heterogeneity of the ND surface. These combined effects can reduce the effective electric charge, or zeta potential, of the ND surface, thereby reducing ND stability in solution.

The detonation nanodiamonds (DNDs) we use in this study have heterogeneous surfaces as a result of their explosive detonation synthesis^19^. These heterogeneities include non-polar fullerene-like reconstructions (FLRs^20,21^), negatively charged polar moieties such as carboxylic acid groups and anhydrides, and positively charged polar moieties such as hydroxyl groups, hydrogen, and amino groups^22^. DNDs thus aggregate readily in most solutions unless they are first treated with a homogenizing surface functionalisation (e.g., C–H, C–OH, COOH, C–F terminations)^23–30^. Beyond direct modification of the DND surface, various other methods to homogenize the surface of DND have been developed. These commonly include coating DNDs with self-assembled monolayers (SAMs) made of polymeric compounds such as oxysilanes and other organic molecules (e.g. diazonium salts, polyglycerol, etc.^23,28,29,31–33^). Of these SAM methods, silanization of nanodiamond^23,29,31^ has proved popular, despite its drawback of low-pH hydrolysis degradation. This popularity can be ascribed to its widespread adoption in other nanoparticle systems^34,35^, the large library of commercially available functional oxysilanes, and its simple sol-gel condensation chemistry that makes it readily accessible to non-chemist practitioners.

While many silanes can be employed to DND functionalisation (e.g. azide functional silanes^36^), the main silane used for ND functionalisation to date has been APTES^23,29^, which coats the ND surface with a positively charged amino group that can subsequently be derivatised. APTES-coated nanoparticles are unstable in biological media because the constituents of media are predominantly negatively charged, although the particles can be made biocompatible by functionalising their surfaces with saturating levels of negatively charged load molecules. This approach is typically not straightforward because the negatively charged load molecules can induce aggregation of the positively charged APTES-coated nanoparticles during functionalisation.

In this paper we present a method for producing monodispersed, soluble DND-biomolecule conjugates using silanisation. We combine two silanes in a modified Bead-Assisted Sonication Disintegration (BASD) process^23,29,31^ to impart DNDs with a negative zeta potential that is maintained throughout subsequent functionalisation and bioconjugation processes to obtain monodispersed DND-biomolecule conjugates with small size. Mixed Silane BASD (MSBASD) builds on and simplifies the BASD process, and enables the addition of multiple functional groups to the DND (or ND) surface via *in situ* silanization in water (substituting previously used anhydrous solvents). With MSBASD, one silane of strong negative charge is added in majority, and the other silane(s) is added in the minority and provides an orthogonal functional group that can subsequently be targeted for conjugating to biomolecules.

Herein we demonstrate MSBASD by functionalising DND with two silanes that are used to similar effect for silica nanoparticles^37^: 3-(trihydroxysilyl)propyl methylphosphonate, monosodium salt solution (THPMP) imparts a negative zeta potential to the DND, and (3-aminopropyl)triethoxysilane (ATPES) provides an amino group for subsequent derivatisation. Using this approach we generate DND conjugates with fluorescein isothiocyanate (FITC) dyes and single-stranded DNA oligomers that are small in size and well dispersed in solution. Given that silanization using a variety of silanes^29,31,36^ is an effective functionalisation approach for various types of hydroxylated ND materials^31,36^, MSBASD is a potentially versatile method for the dispersion and conjugation of nanodiamond materials for solution-based applications.

## Experimental Methods

All data generated or analysed during this study are included in this published article (and its Supplementary Information files).

### Materials

DND powder (NanoAmando, dry powder form) was purchased from New Metals and Chemical Ltd. (Tokyo, Japan). Unless stated otherwise all chemicals were reagent grade and purchased from Sigma Aldrich. De-ionised (DI) water was used for all reactions and washing steps.

### MSBASD Apparatus

**Figure 1:**
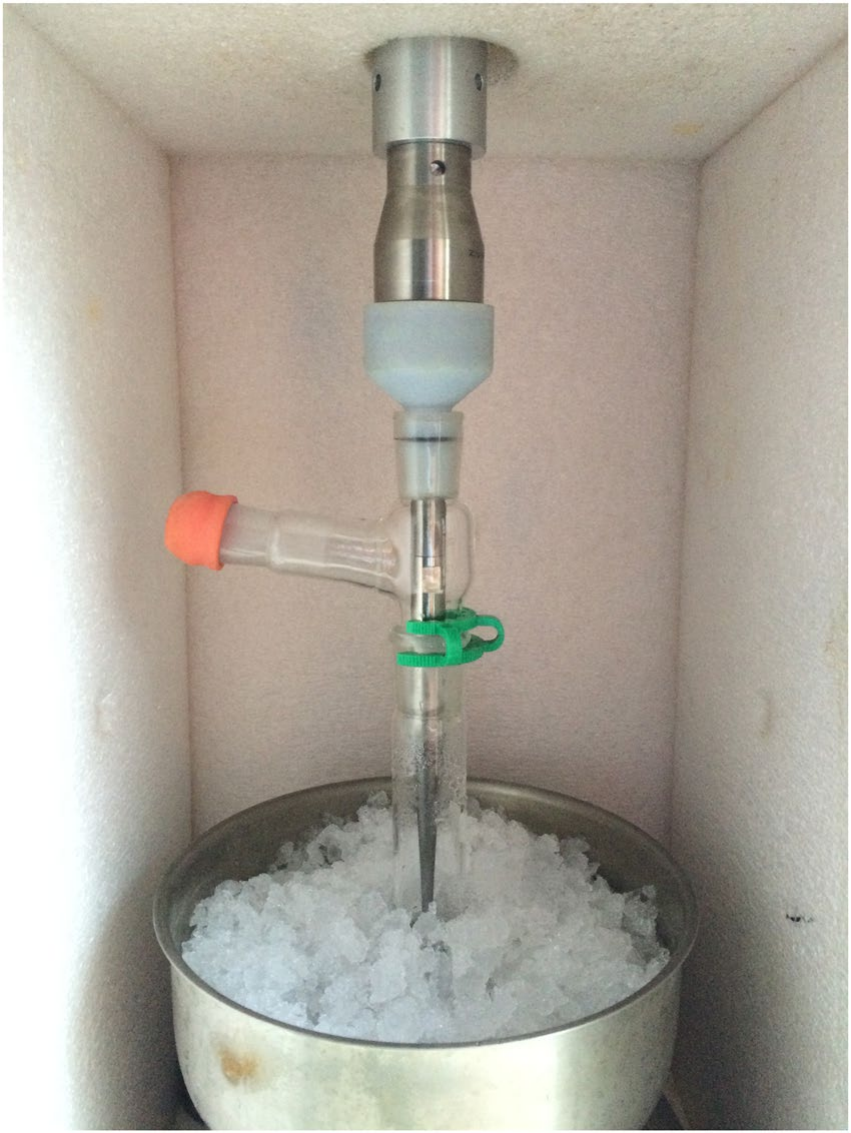
MSBASD apparatus with 3D printed adapter.

The MSBASD apparatus was constructed using standard chemical glassware pieces and a custom 3D printed adapter (3DPA) (see Supplementary Information for.stl file). The 3DPA hermetically seals the glassware (B19 19/26 joint) with the sonicator probe (Vibra-Cell VXC500) around the null-point of vibration. The 3DPA was printed on an Objet30 printer in VeroGray material and contains two *o*-rings (RS Components, ×2 BS220 SIL70 & BS112 NBR) to seal onto the ultrasonic probe and glassware. A custom adapter was used instead of a commercial sonicator reaction vessel because most laboratory sonicator probes are not screw threaded probes, which are required to connect with commercial reactor vessels. 3DPA was designed so that the accompanying glassware was positioned optimally with respect to the 3 mm tapered probe. This glassware consisted of a standard 2 or 3 neck adapter (e.g. Fisher cat. #:11312035 3 neck multiple adapter glassware) with a topside central B19 (19/26 female) connector and lower central B24 (24/29 male) connector, to which a 150 mL test tube with B24 (24/29 female) connector was connected (Fisher cat. #:11342055). The reaction test tube was held in an ice bath for cooling during sonication. A picture of the apparatus using a 2-neck multiple adapter is shown in Fig. 1.

### DND–OH synthesis

**Figure 2:**
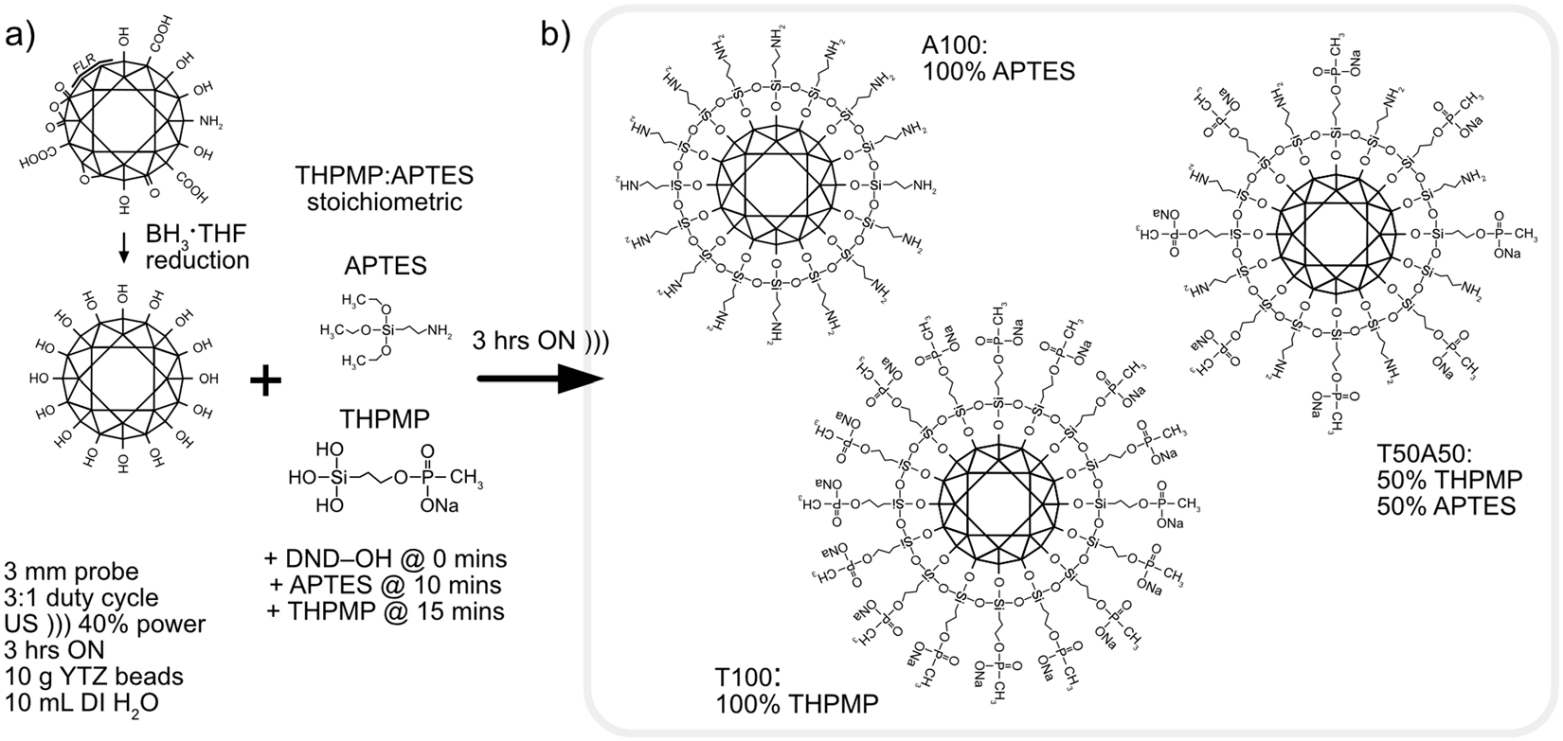
(**a**) Silanization reaction scheme and (**b**) example DND TA products (idealized representations).

DND–OH was synthesised as depicted in Fig. 2a following the protocol in Krueger *et al*.^29^. First (2.33 g) DND powder was dried under rotary evaporation in toluene to yield (~2 g) dry powder (14% H_2_O mass). (2 g) of dry DND powder was suspended in (120 mL) anhydrous tetrahydrofuran (THF) under a dry N_2_-vented reflux apparatus. 1 M BH_3_·THF (SigmaAldrich cat. #:176192) (20 mL) was added dropwise with stirring to the suspension and heated under reflux for 24 h^29^. Once cool, the solution was neutralised by dropwise addition of 2 M aq. HCl until bubble production ceased. The DND–OH product was washed 4 times with DI water followed by 10 times with acetone using the following 3-step process cycle: (1) centrifugation to form a sample pellet (70 k × g RCF, 4 mins), (2) supernatant replacement with fresh solvent and (3) ultrasonic (US) resuspension using a Sonics Vibra-Cell VXC500 equipped with a 3 mm tapered probe (3:1 duty cycle 40% power, 3 mins or until dispersed). Washes in acetone instead of water were required to pellet the DND sample after the first few DI water wash cycles. A final wash with DI water was performed with the supernatant reaching a neutral pH 7. The sample was dried under rotary evaporation and stored in a vacuum desiccator until use.

### Silanization functionalisation

For each functionalized DND preparation, (100 mg) of DND–OH and (10 g) YTZ 50 µm Yttrium Zirconia milling beads (Tosoh Ltd.) were added to a test tube with (10 mL) DI water and sonicated at 40% power, 3:1 duty cycle, for 10 mins to redisperse dry powders. For silanization, APTES (99%, SigmaAldrich cat.#:440140) and THPMP (50% in H_2_O, SigmaAldrich cat.#:435716) were added in different amounts to give the desired THPMP:APTES ratio, hereafter given in the notation of T*P*A*N*, where *P* and *N* are substituted for the percentages of THPMP and APTES respectively (Fig. 2b). APTES was added in volume *N* * (1.1 µL) (e.g. for 10% APTES, (11 µL) APTES was added). THPMP was added in volume *P* * (3 µL). The solution was then sonicated for a total of 4 h (40% power, 3:1 duty cycle). APTES was added 5 mins prior to THPMP to allow hydrolysis of the less liable alkyloxy group of APTES^38^. Following sonication the sample was left to settle for 5 minutes, after which the black sample supernatant was removed and the remaining sample retrieved from the settled YTZ beads by rinsing with acetone. The T*P*A*N* samples were washed 5 times in (50 mL) acetone with the above 3-step process cycle, except using (50 k × g RCF, 4 mins) centrifugation. A final wash cycle using DI water was used to redisperse the sample. To remove YTZ debris and large particles, samples were centrifuged for 5 mins at 5 k × g RCF. The supernatant was lyophilized in (1 mL) aliquots (~12–14 aliquots total) and either stored under vacuum to retain amino reactivity, or used immediately for derivatisation. Prior to subsequent use dried samples were re-dissolved using US and centrifuged at (5 k × g RCF) to remove large particles.

### FITC conjugation

Excess *N*-hydroxy-succinimidyl ester fluorescein (NHS-FITC) dye (ThermoFisher Scientific cat. #:46410) was added to T80A20 DND (1 mg/mL) in 1 mM phosphate buffered saline (PBS) and mixed for 30 minutes at room temperature. FITC-T80A20 was washed using a 50 kDa MWCO centrifugation filter (50 kDa MWCO Amicon Ultra Ultracel Amicon, 2 k × g, 10 minutes, 3.5 mL wash DI water) until the waste wash contained no trace of FITC (as determined visually and by NanoDrop UV-visible spectroscopy).

### Sulfo-SMCC conjugation

~30 mg (4 aliquots) of lyophilized T80A20 was added to (8 mg) of Sulfo-SMCC (sulfosuccinimidyl 4-(*N*-maleimidomethyl)cyclohexane-1-carboxylate, ThermoFisher Scientific cat. #:22622, 2 mg × 8 no-weigh format) dissolved in (2 mL) of 1 mM PBS and mixed for 30 mins at room temperature to generate maleimide-activated T80A20 (Mal-T80A20). Samples were washed with 50 kDa MWCO centrifugation filtering (Amicon Ultra-4 50k NMWL, ×3 washes, 2 k × g RCF, 10 mins, 3.5 mL wash DI water). Approximately 1 ml of mal-T80A20 in DI water (estimated ~20 µM) was recovered and then frozen in (50 µL) aliquots at −80 °C to preserve maleimide activity.

### DNA conjugation

A 3′-thiol-C3-modified (disulphide) single-stranded DNA oligomer (ssDNA, Integrated DNA Technologies, 73 nucleotides, see Supplementary Information) was added in 10-fold excess to mal-T80A20 by mixing (10 µL) of 20 µM mal-T80A20 (200 pmoles) with 2 nmoles of ssDNA in 1 mM PBS, (5 mM) TCEP·HCl (ThermoFisher Scientific cat. #:20490) in a (100 µL) reaction volume. The reaction was mixed for at least 30 mins and washed with 50 kDa MWCO centrifugation filtering (x5 washes, 2 k × g, 10 mins, 3.5 mL wash 1 mM PBS). Wash effluent was collected and inspected for unbound ssDNA by measuring absorbance at 260 nm using NanoDrop UV-visible spectroscopy. Washes were continued until there was no unbound DNA detected in the wash effluent for three consecutive washes.

### Characterisation

Fourier Transform Infrared Spectroscopy (FTIR) was performed using a Bruker Vertex 70 FTIR equipped with a Germanium attenuated total internal reflectance (ATR) accessory. For FTIR measurements, nanoparticle powders were suspended in methanol and drop-dried onto the ATR crystal. Prior to analysis, FTIR spectra were processed with ATR correction, baseline correction, and, for plotting, normalisation. Classical Least Squares (CLS) deconvolution was performed using the Normal Equation, *W = (X*^*T*^*X*)^*−1*^*X*^*T*^*D*, where *D* is a matrix of FTIR spectra, *X* is a matrix of A100 and T100, and *W* is a matrix giving the weights of deconvolution for A100 and T100. For plotting, deconvolution weights were normalized to a sum of 1. The linear fit to CLS deconvolution (%) W_A100_ / (W_A100 +_ W_T100_) vs. silane precursor *N/(N* + *P)* was performed using MATLAB (Levenberg-Marquardt algorithm). Pearson’s r = 0.96, p = 5.93 × 10^−4^, m = 0.93, c = 12.7%.

X-ray Photoemission Spectroscopy (XPS) was performed using a ThermoScientific K-Alpha X-ray Photoelectron Spectrometer equipped with an Al K-alpha source. XPS analysis and peak fitting was performed using MATLAB as described in Supplementary Information.

To estimate the nitrogen contribution from APTES (*N*_*APTES*_) vs. intrinsic DND nitrogen (*N*_*DND*_) in the N1s XPS measurement (N1s peak integration: *N*_*XPS*_), a model was constructed as described in Supplementary Information. A model was used in place of N1s peak deconvolution due to the weak signal and strong overlap of *N*_*DND*_ and *N*_*APTES*_ contributions to *N*_*XPS*_. The model transforms *N*_*XPS*_ into *N*_*APTES*_ as follows: *N*_*APTES*_* = N*_*XPS*_ − *0*.*0153C*_*XPS*_, where 0.0153 corresponds to an estimated fractional percentage of nitrogen relative to carbon (*C*_*XPS*_) which is intrinsic to DND (*N%*_*DND*_). The *N%*_*DND*_ fit of 1.53% shows reasonable agreement to expected DND nitrogen percentages (2–3%)^39^ and good agreement to the herein measured 1.67% of T100 and DND–OH samples. The linear fit to *N*_*APTES*_/(*N*_*APTES*_ + *P*_*XPS*_) vs. silane precursor *N/(N* + *P)* was performed using MATLAB (Levenberg-Marquardt algorithm) and a fit weighting of ${N}_{{SNR}}^{-2}$, where ${N}_{{SNR}}$ is the signal-to-noise estimation of N1s peak integration (*N*_*XPS*_) for each XPS measurement. Pearson’s r = 0.98, p = 1.13 × 10^−4^, m = 0.93, c = 5.3%.

**Figure 5:**
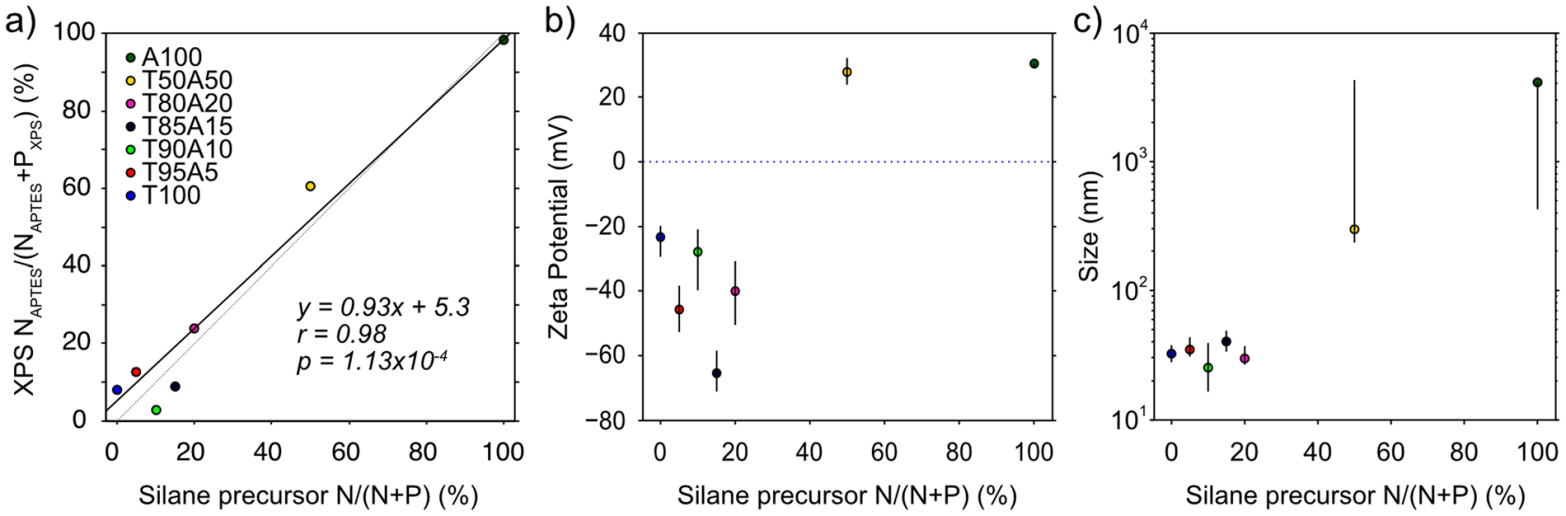
(**a**) XPS-inferred *N*_*APTES*_*/(N*_*APTES*_ + *P*_*XPS*_*)* percentage vs. silane precursor reagents *N/(N* + *P)*%. The dotted line corresponds to an exact linear relationship between the variables. The solid line shows a linear fit to the data. A general linear fit is observed with a Pearson**’**s r (0.98) & p (1.13 × 10^−4^) values inset. (**b**) Zeta potential and (**c**) Size distributions of TA samples vs. silane precursor reagents *N/(N* + *P)*%. For size and zeta values, data points represent median values and error bars are upper and lower quartiles.

Dynamical Light Scattering and Zeta potential measurements were performed using a Zeta NanoSizer ZS, Malvern Instruments, (DTS1070 capillary cells) in 0.1 µm syringe-filtered DI water in backscatter configuration (633 nm laser 173°) using a bulk ND refractive index of 2.4. Data points in Fig. 5b and 5c for size / charge correspond to median distribution values and error bars correspond to the upper and lower quartile values of the distributions.

Gel electrophoresis was performed using pre-cast 0.8% agarose gels (E-gel, ThermoScientific) and 1 kbp DNA ladder (N3232S, New England Biosciences). Gel images were captured using a Gel Doc EZ system.

High Performance Liquid Chromatography (HPLC) was performed using a Waters 600 HPLC system (Waters 2487 Dual Wavelength Absorbance Detector) and a Size Exclusion Chromatography (SEC) column (Sepax SRT-C 300 Å 5 µm 4.6 × 50 mm column). 0.2 µm vacuum–filtered degassed (US-assisted) DI water was used as the mobile phase using 1 mL/min flow rate and monitored at 260 and 300 nm absorption wavelengths.

Atomic Force Microscopy (AFM) was performed using a JPK Nanowizard in liquid tapping mode using SiN 0.06 N/m probes in 10 mM HEPES buffer on cleaved MICA. AFM data was preprocessed using median line correction, singular value decomposition flattening and median value kernel filtration in MATLAB prior to analysis. Particle height was measured by threshold detection (>2 nm) and subtracting the maximum particle height from the surrounding MICA surface height for each particle.

## Results and Discussion

Long-term disintegration and suspension of DND has several diverse challenges, all of which stem from their explosive synthesis^19,40^. The detonation process leaves DNDs in an unusually tight aggregated form^25^, bound together by sooty remnants. These aggregates must be atomically homogenized (e.g., with hydrogen^24,41^ or oxygen^27,29,42,43^) and disintegrated with high power ultrasonic processing^23,26^ to achieve monodispersed hydrocolloid suspensions. The well-dispersed nature of these suspensions can be fleeting, however, due to unwanted effects of the ultrasonic processing step on the DND surface. Ultrasound (US) can change the surface chemistry of DNDs by introducing hydroxyl groups and FLR regions^28^. These changes can reintroduce heterogeneity to the DND surface and cause dispersed solutions to gradually re-aggregate. It is therefore advantageous to use hydroxyl termination of the DND surface as both the homogenizing disintegration step and the foundation for subsequent functionalisation.

Thus, to functionalise DNDs we chose a hydroxyl-based sol gel chemistry approach, first homogenising the DND surface with hydroxyl groups from BH_3_·THF reduction and then functionalising the surface with derivatisable oxysilanes (see Experimental Methods). To improve the monodispersion of silanized DNDs, we have used *in situ* silanization with BASD processing^31^ to fully disperse and immediately functionalise DNDs with self-assembled silanes before they can re–aggregate. MSBASD extends the BASD process by mixing two (or more) silanes with the desired stoichiometry prior to the reaction step to allow tailored, functional composition of the DND surface. Furthermore, to simplify the reaction process we have modified MSBASD so the procedure can be performed in DI water instead of anhydrous organic solvent^31,35^. The relaxed reaction conditions are possible because the BASD process acts to prune and hinder inter-particle bonding and reduce the build-up of multiple silane layers that are normally ameliorated via anhydrous conditions.

We demonstrate the MSBASD process by producing highly soluble FITC-DND and DNA-DND conjugates using two functional silanes. THPMP, an oxysilane with a negatively charged functional group, aids solvation of DNDs while the commonly used APTES provides an amino group for functionalization. Using mixtures of THPMP and APTES with different stoichiometries, with THPMP in majority^37^, we obtain monodispersed, negatively charged, soluble DNDs with a defined number of functional groups for subsequent grafting of chemical modifications or biomolecules.

### Mixed silanization functionalisation

**Figure 3:**
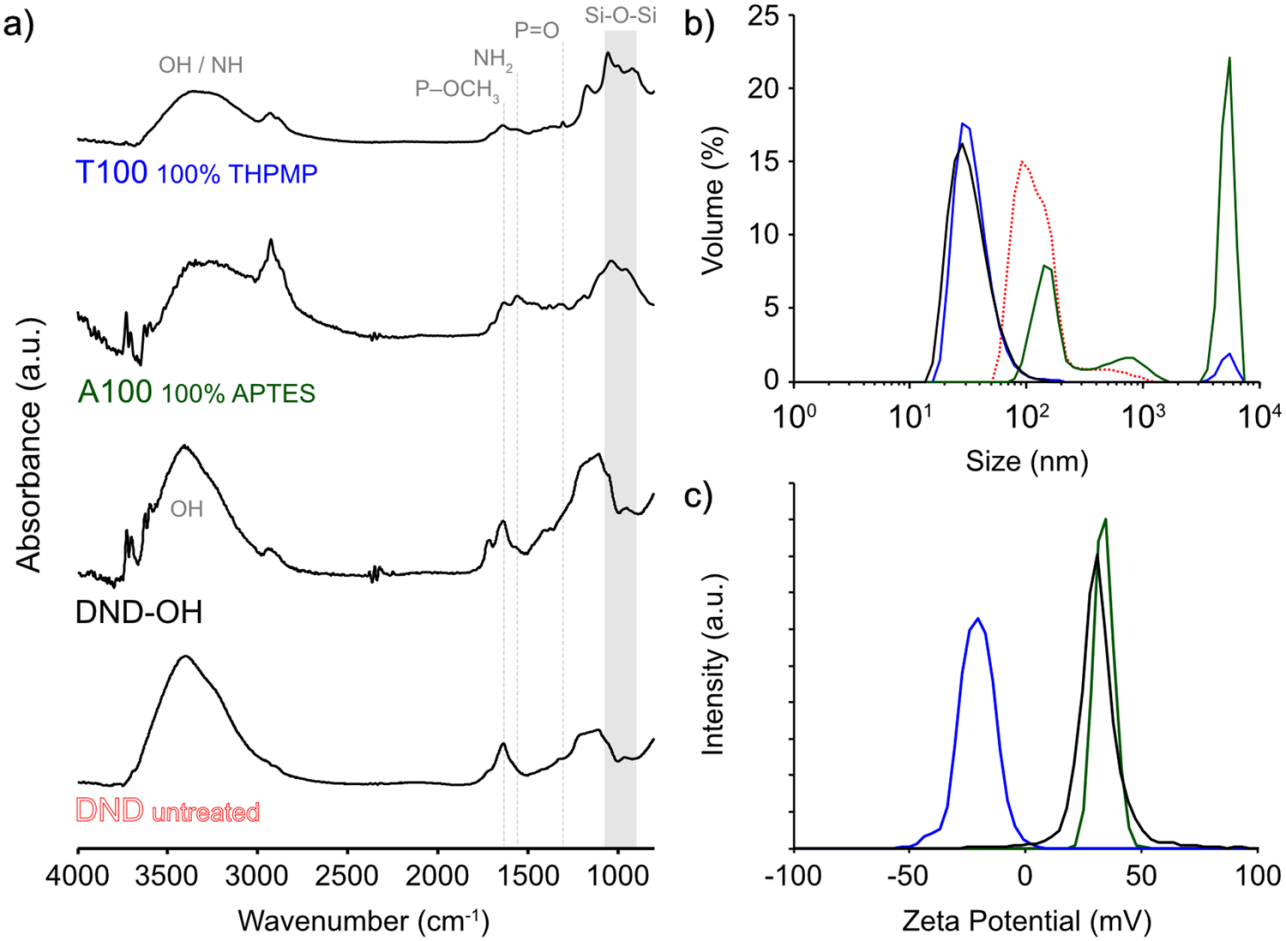
(**a**) FTIR spectra of DND materials and APTES/THPMP functionalised DND, with salient peaks indicated in grey. (**b**) DLS hydrodynamic size and (**c**) zeta potential distributions of panel (a) samples.

Following the preparation of DND–OH, we first silanized DNDs using either APTES or THPMP, using the modified BASD process as described in Experimental Methods. Figure 3a shows the FTIR spectra of DND before (untreated DND and DND–OH) and after silanization using 100% APTES (A100) and 100% THPMP (T100) in water. Relative to the DND-OH spectrum, the spectra for both A100 and T100 showed a reduction in the intensity of the OH stretching mode (broad peak between 3200 to 3700 cm^−1^), as expected in the condensation reaction.

For both A100 and T100 samples, DND silanisation was confirmed by strong siloxane (Si–O–Si) stretching vibrational modes observed at lower wavenumbers between 900 and 1050 cm^−1^. The peaks were redshift in comparison to equivalent bulk surface values as observed previously for silanized BASD-processed DND^31^. This redshift is due to the very small particle size of BASD-processed DND, whereby lower wavenumber vibrational modes are redshift in frequency due to their larger reduced mass on lighter mass substrates^44^.

For A100, APTES functionalisation was confirmed by the characteristic NH_2_ bending mode at 1557 cm^−1^, corresponding to the APTES functional head group^45^. For T100, THPMP functionalisation was confirmed by identification of two peaks corresponding to head group moieties of THPMP^46^. One peak sharply centred at 1306 cm^−1^ was ascribed to phosphine oxide (P = O) stretching, and a second peak centred at 1643 cm^−1^ was ascribed to methylphosphonate (P–OCH_3_). While the methylphosphonate can be distinctly assigned to THPMP^46^ and T100, this wavenumber is heavily convolved with the OH bend vibrational mode of absorbed water (~1640 cm^−1^) and therefore its presence in other samples is not necessarily ascribed to methylphosphonate.

Having established silanization of DND using APTES and THPMP, the hydrodynamic size and zeta potential of samples was determined. Figure 3b shows the DLS size distributions for each sample. The size distribution of as-purchased, untreated DND (monodispersed size ~ 5 nm) had a median value of 103 nm. This aggregated size is considerably larger than the minimum monodispersed form of DND. DND–OH showed a reduced size of 27 nm following BH_3_·THF treatment with a zeta potential of +28 mV (Fig. 3c), as expected for hydroxylated DND^23^. BASD silanization for A100 resulted in a slightly increased and well-defined zeta potential of + 30 mV, however, aggregation was evident with multimodal-sized aggregates and a median size of ~4 µm. T100 showed improved dispersion behaviour with a negative zeta potential of −23 mV and a sharp size distribution median of 30 nm, in good comparison to the small size achieved without *in situ* silanization.

**Figure 4:**
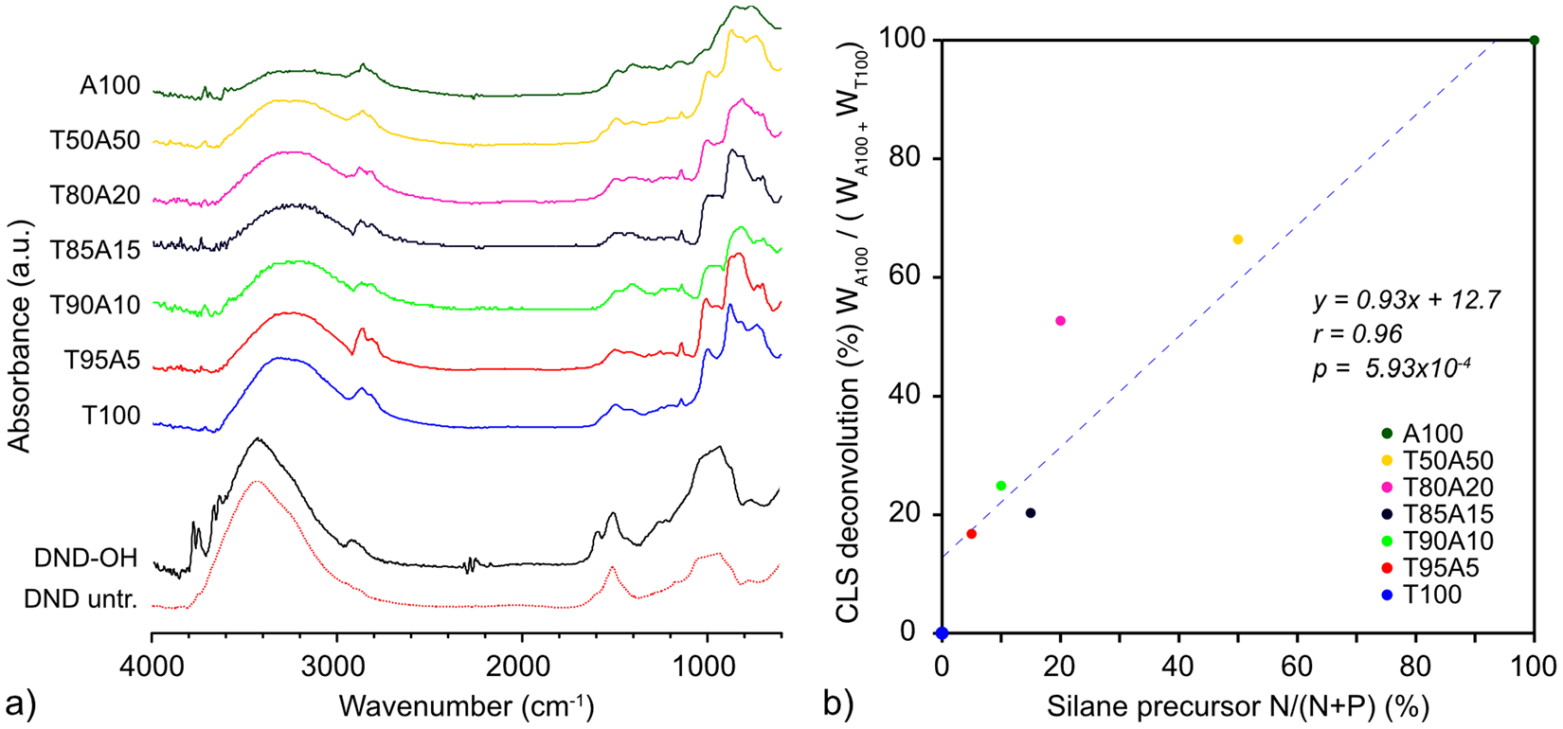
(**a**) Normalized FTIR spectra of TA variation samples with arbitrary offset. (**b**) CLS deconvolution of A100 and T100 spectra from TA spectra vs. N/(N + P)%. The blue dashed line shows a linear fit of the data with a Pearson**’**s r (0.96) & p (5.93 × 10^−4^).

Having established that THPMP silanization produces negatively charged, well-dispersed DND, the reaction ratio of THPMP:APTES (TA) was varied in order to determine a range of suitable TA stoichiometries that produced well-dispersed, negatively charged, derivatisable DND. Figure 4a shows the FTIR spectra of tested TA combinations. In an attempt to quantify the amount of APTES and THPMP bound to TA samples via FTIR spectra, a CLS deconvolution of A100 and T100 spectra from all TA spectra was performed, as described in Experimental Methods (Fig. 4b). Despite the shortcomings of the efficacy of CLS deconvolution using variable FTIR measurements (e.g. variations in baseline, atmosphere, absorbed water, sample preparation, etc.), a roughly linear relationship was observed for the percentage of A100 deconvolved vs. nominal APTES percentage.

In order to better estimate the ratio of bound THPMP vs. APTES, XPS Spectroscopy was used to measure the ratio of nitrogen (1 N in APTES) to phosphorous (1 P in THPMP) in each sample. Figure 5a shows the estimated *N*_*APTES*_*/(N*_*APTES*_ + *P*_*XPS*_*)%* vs. the nominal stoichiometric *N/(N* + *P)%* of THPMP and APTES. A general linear trend between the nominal and measured TA stoichiometry was observed that, along with the FTIR CLS deconvolution (Fig. 4b), suggested that DND functionalization with the desired silane stoichiometry was achieved within reasonable margins.

Having established estimates for the THPMP vs. APTES ratios bound to the DND, DLS was used to determine the hydrodynamic size and charge of TA samples with respect to silane precursor stoichiometry. Figure 5b shows the median zeta potential of all TA samples vs. precursor N/(N + P)% in DI water (pH 7). Although there was not a clear correlation between zeta potential and silane stoichiometry, as the percentage of APTES increased the zeta potential switched from negative (T100) to positive (A100) as expected. This transition occurred between T80A20 and T50A50 samples. Of note, for small APTES percentages, an increase in the magnitude of the negative zeta potential in comparison to T100 was observed, as also observed for silica nanoparticles^37^. The nonlinear relationship of zeta potential with respect to TA ratio and in particular the negative zeta potentials at ≤20% APTES percentage suggests that, at low APTES percentages, DND can be loaded with functional amino groups without diminishing the strong negative zeta potential imparted by THPMP. Increasing the APTES concentration above 20% resulted in a large increase in hydrodynamic size of the TA samples (Fig. 5c), suggesting that their increased zeta potential was increasing aggregation of the DNDs.

In light of these measurements, we chose 20% APTES DND (T80A20) for chemical ligation. While any of the 5–20% APTES samples would be suitable for subsequent conjugation, T80A20 was selected due to its particularly strong negative zeta potential (−40 mV), its large number of reactive amino moieties, and, foremost, its small hydrodynamic size in comparison to other samples.

### DND functionalization with FITC via amine-NHS chemistry

**Figure 6:**
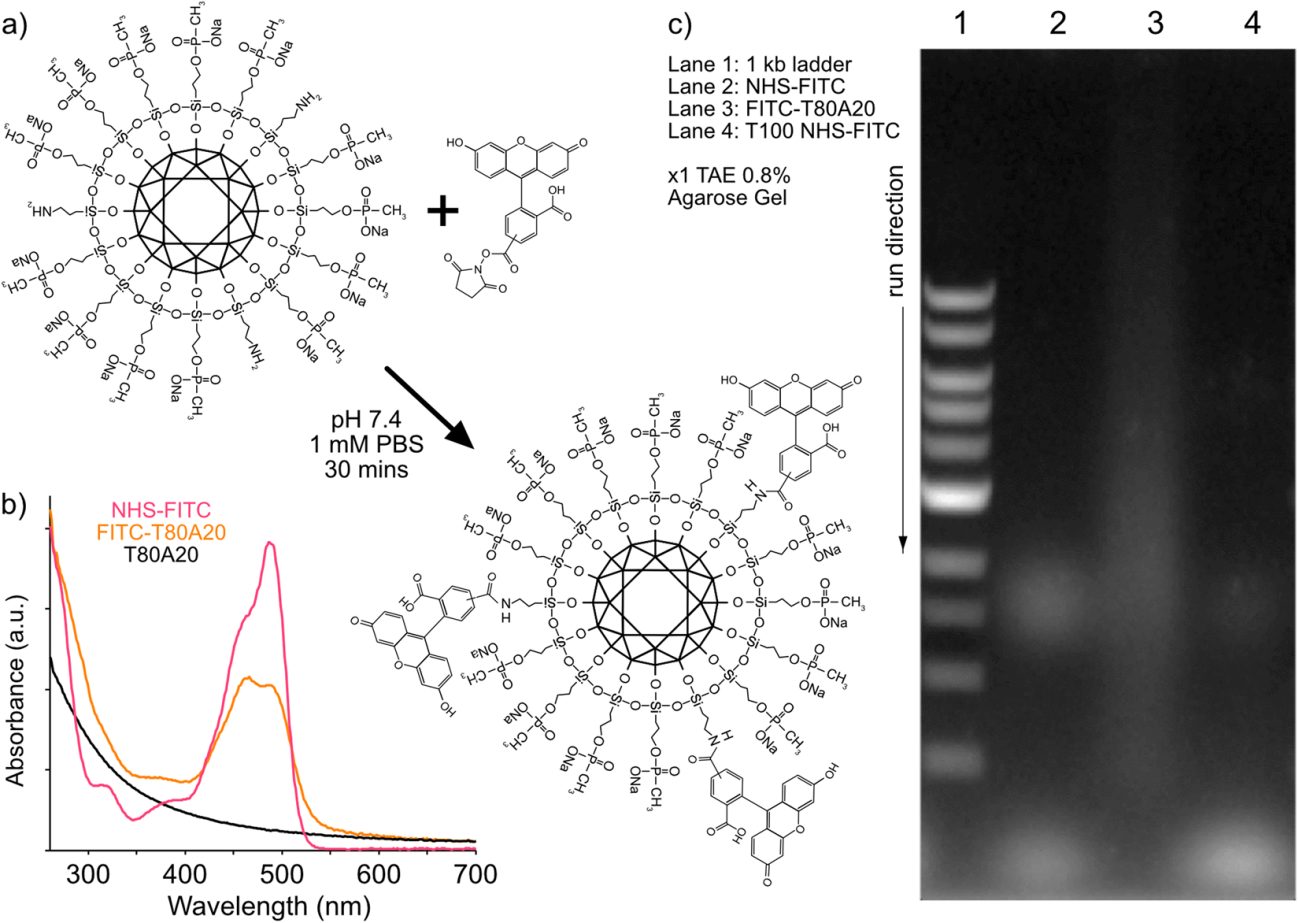
(**a**) Reaction schematic. (**b**) UV-visible spectra of reagents and product FITC-T80A20 conjugate. (**c**) 0.8% agarose gel ×1 TAE running buffer. 3^rd^ lane shows a slower smeared band of FITC dye due to DND conjugation.

To confirm the chemical reactivity of the APTES amino functional groups on the MSBASD TA-functionalized DND, T80A20 was reacted with an amine-reactive *N*-hydroxy-succinimidyl ester fluorescein dye (NHS-FITC), resulting in a fluorescent orange product (Fig. 6a). The FITC-T80A20 conjugation was confirmed using UV-visible spectroscopy (Fig. 6b). To further verify conjugation and therefore amine reactivity of T80A20, we used agarose gel electrophoresis to demonstrate the slower transit of the FITC-T80A20 in comparison to unbound FITC-NHS. Figure 6c demonstrates that FITC-T80A20 (3^rd^ lane) migrates in a smeared slow band in comparison to the unbound NHS-FITC (2^nd^ lane) and unconjugated mixture of T100 and NHS-FITC (4^th^ lane). This is, to our knowledge, the first demonstration of DND migrating in an electrophoresis gel. No conjugation was expected for the sample in lane 4 due to the lack of amino groups on T100, which confirms APTES amino groups were responsible for and capable of amine-NHS reactions on T80A20 NPs.

### DND functionalization with DNA via maleimide-thiol chemistry

Having established that T80A20 was accessible and reactive with NHS groups, we next sought to conjugate T80A20 to single-stranded DNA oligomers using the heterobifunctional crosslinker Sulfo-SMCC. Sulfo-SMCC has an NHS-reactive group and a thiol-reactive maleimide group. The NHS moiety of Sulfo-SMCC was first reacted with the amino groups on T80A20 to generate maleimide-activated T80A20 (mal-T80A20). Mal-T80A20 was then reacted with thiol-modified DNA to produce DNA-T80A20 conjugates. Reactions were performed as described in Experimental Methods. While both reactions can be performed in immediate succession, in this study we first reacted Sulfo-SMCC with T80A20 at a large scale and stored it in aliquots as a dry powder at −80 °C to preserve maleimide reactivity. The FTIR spectrum of mal-T80A20 is shown in 7c.

**Figure 7:**
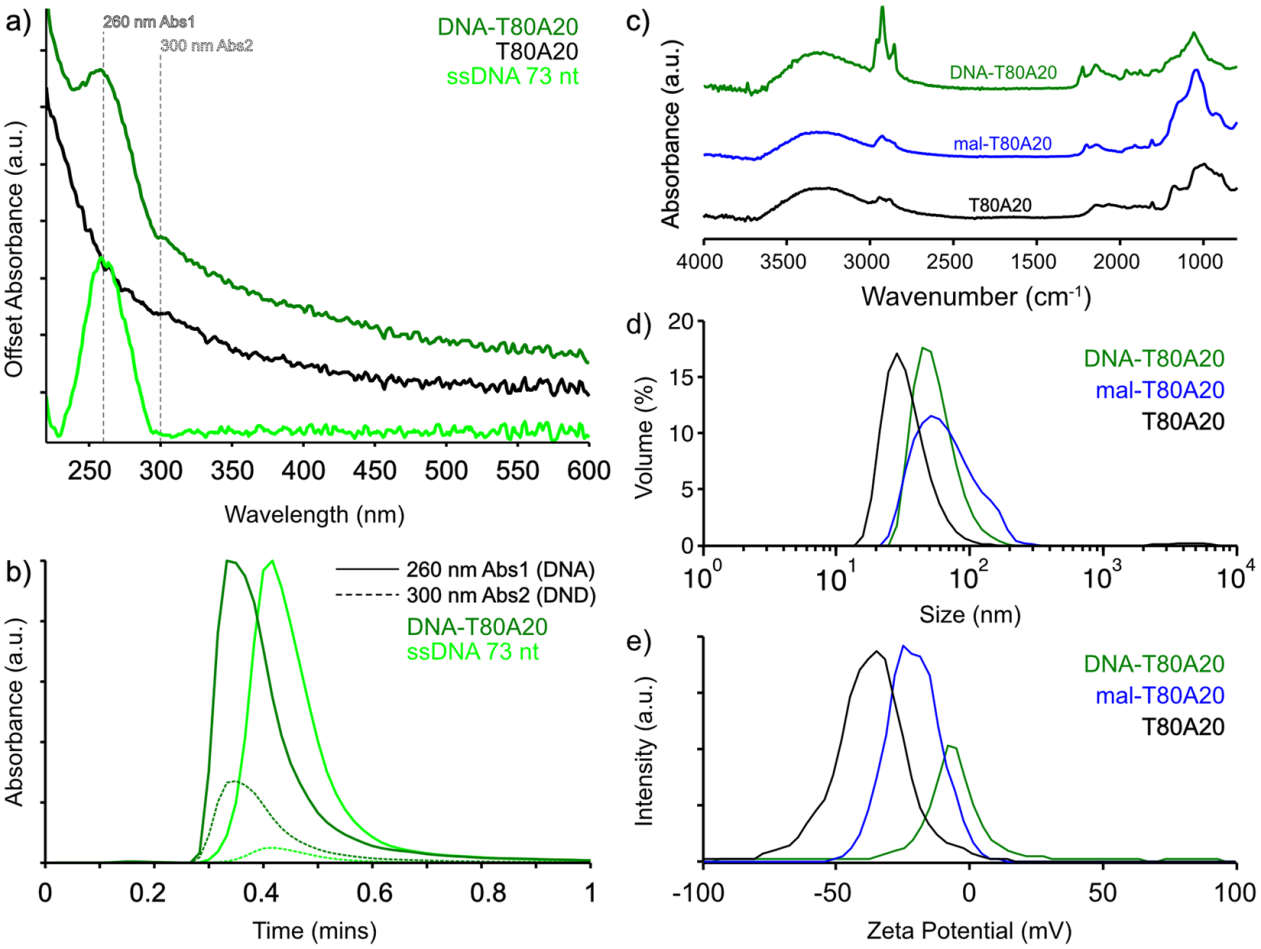
(**a**) UV-visible spectra of DNA-T80A20 conjugate and precursors. (**b**) HPLC of two separate time-aligned runs of DNA and DNA-T80A20. Solid lines show absorbance at 260 nm, dashed lines at 300 nm. Absorbance is normalized to the maximum 260 nm absorbance for each sample. (**c**) FTIR spectra of DNA-T80A20 conjugate, reaction intermediate mal-T80A20 and T80A20. (**d**) Hydrodynamic size and (**e**) zeta potential distributions of DNA-T80A20, mal-T80A20, and T80A20 conjugates.

We next conjugated thiolated, single-stranded DNA oligomers (ssDNA) to mal-T80A20. UV-visible spectroscopy was used to verify the presence of both DNA and T80A20 in the collected product. Figure 7a shows the UV-visible spectra of T80A20, ssDNA and the DNA-T80A20 conjugate. The broad peak at 260 nm of DNA-T80A20 in comparison to the sloping spectrum of T80A20 confirmed the presence of both DNA and T80A20 in the retained product. Similarly, the FTIR spectra in Fig. 7c confirmed DNA and T80A20 to be present in the product, as verified by the presence of the following peaks: a convolved peak at 1058 cm^−1^ corresponding to backbone PO^−^_2_ stretching and furanoside CO stretching^27,47^; and ring stretching modes^27^ between 1200 and 1700 cm^−1^ including C = C and C = N ring vibrations of adenine at 1647 cm^−1 47^ (adenine being 58% of base pairs in ssDNA used herein). Covalent conjugation of ssDNA and T80A20 was subsequently verified using High Performance Liquid Chromatography (HPLC) Size Exclusion Chromatography (SEC) as described in Experimental Methods (Fig. 7b). Two absorbance wavelengths (260 and 300 nm) were monitored simultaneously in order to distinguish DNA-T80A20 from ssDNA via their absorbance ratios, as indicated by the dashed lines in Fig. 7a. From separate time-aligned runs, HPLC measurements showed DNA-T80A20 to pass through the column more quickly than the smaller unbound ssDNA (as expected for SEC), confirming DNA conjugation to T80A20.

**Figure 8:**
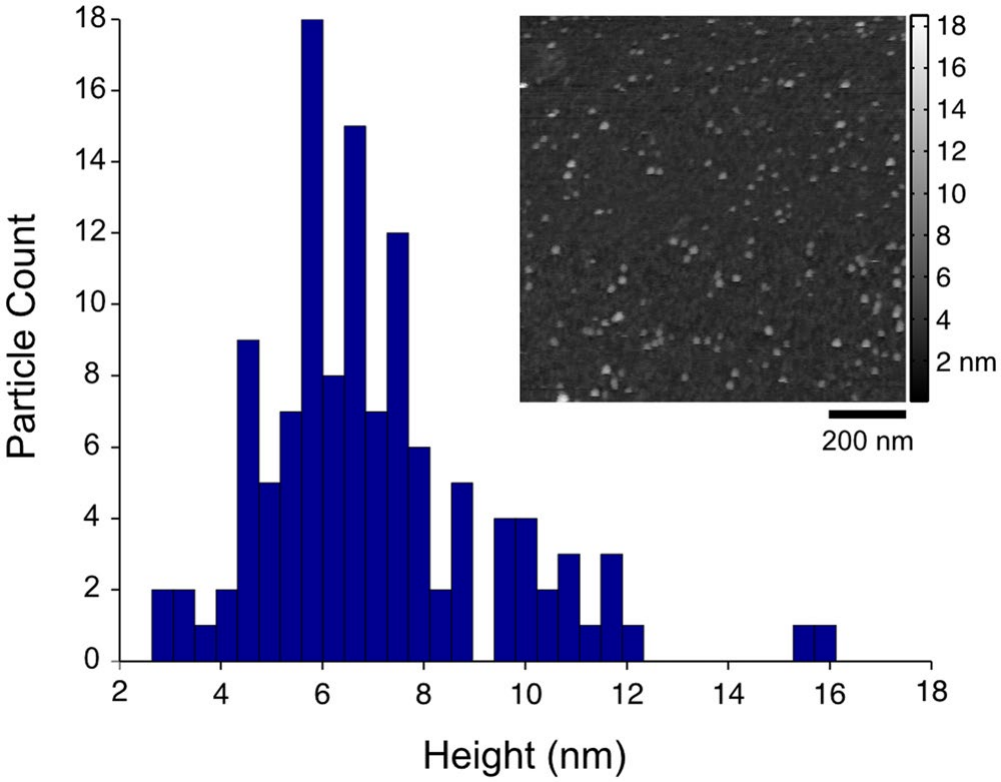
Histogram of DNA-T80A20 particle height as determined by AFM with inset corresponding image (1 µm^2^ area). Particle heights indicate monodispersed DND conjugates.

Figure 7d shows the size distributions of T80A20, mal-T80A20, and DNA-T80A20. Mal-T80A20 was observed to aggregate slightly, but subsequent conjugation with ssDNA eliminated this aggregation, resulting in a redispersion of the conjugate. The conjugate size of median 46 nm and mean 56 nm is substantially smaller than previously reported biomolecular conjugates^29^ made using APTES silane chemistry, which were ~300–500 nm in size, and similar to PNA-DNA conjugates^48^ (mean 44 nm). To further probe the physical conjugate size (as opposed to larger hydrodynamic size), AFM was used to measure the height of conjugates as described in Experimental Methods. Figure 8 shows a histogram of the measured heights of DNA-T80A20 to be centered at ~6 nm, in good agreement with sizes expected for monodispersed DND. This result shows MSBASD to be effective at monodispersing and functionalizing DND particles. Finally, the zeta potential of the DNA-T80A20 were sharply centered at weak zeta potential values of −9 mV (Fig. 7e) and showed an aggregation resilience in divalent cationic media (MgCl_2_) up to ~8 mM concentration (Figure S1).

## Conclusions

The Mixed Silane Bead-Assisted Sonication Disintegration (MSBASD) process presented in this paper extends and modifies the *in situ* silanization BASD process. MSBASD is a versatile and accessible functionalisation approach for producing detonation nanodiamond (DND) nanoparticles that are derivatisable, have (weak) negative zeta potential, are monodispersed, and are highly soluble. Importantly, MSBASD enables the addition of a defined number of chemical and/or biological functional groups to the DND surface. We believe that MSBASD will be of particular interest to those wishing to synthesize monodispersed nanodiamond conjugates that are decorated with multiple probes and/or biomolecules. The examples shown herein of DNA-DND and FITC-DND conjugates employ the mixed silanes of APTES (20%) and THPMP (80%) to achieve the smallest conjugates assembled to date, with DNA-DND conjugates having a hydrodynamic size of ~50 nm and AFM height of ~6 nm.

## Electronic supplementary material

Supplementary Information

MSBASD Adapter

## References

[1] Turcheniuk K, Mochalin VN (2017). Biomedical applications of nanodiamond (Review). Nanotechnology.

[2] Mochalin VN, Shenderova O, Ho D, Gogotsi Y (2011). The properties and applications of nanodiamonds. Nature Nanotech.

[3] Vaijayanthimala V, Tzeng Y-K, Chang H-C, Li C-L (2009). The biocompatibility of fluorescent nanodiamonds and their mechanism of cellular uptake. Nanotechnology.

[4] Schrand AM, Dai L, Schlager JJ, Hussain SM, Osawa E (2007). Differential biocompatibility of carbon nanotubes and nanodiamonds. Diamond and Related Materials.

[5] Liu K-K, Cheng C-L, Chang C-C, Chao J-I (2007). Biocompatible and detectable carboxylated nanodiamond on human cell. Nanotechnology.

[6] Schrand AM (2007). Are Diamond Nanoparticles Cytotoxic?. J. Phys. Chem. B.

[7] Hui, Y. Y., Cheng, C.-L. & Chang, H.-C. Nanodiamonds for optical bioimaging. *J Phys D Appl Phys* **43**, (2010).

[8] Chang Y-R (2008). Mass production and dynamic imaging of fluorescent nanodiamonds. Nature Nanotech.

[9] Yu S, Kang M, Chang H, Chen K, Yu Y (2005). Bright fluorescent nanodiamonds: No photobleaching and low cytotoxicity. J Am Chem Soc.

[10] Merson TD (2013). Nanodiamonds with silicon vacancy defects for nontoxic photostable fluorescent labeling of neural precursor cells. Optics Letters.

[11] Say JM (2011). Luminescent nanodiamonds for biomedical applications. Biophys Rev.

[12] Barnard AS (2009). Diamond standard in diagnostics: nanodiamond biolabels make their mark. Analyst.

[13] Ho D (2009). Beyond the Sparkle: The Impact of Nanodiamonds as Biolabeling and Therapeutic Agents. ACS nano.

[14] Krueger A (2008). New carbon materials: Biological applications of functionalized nanodiamond materials. Chemistry - A European Journal.

[15] Man, H. B. & Ho, D. Diamond as a nanomedical agent for versatile applications in drug delivery, imaging, and sensing. *phys stat sol (a)* **209**, 1609–1618 (2012).

[16] Schrand AM, Hens S, Shenderova O (2009). Nanodiamond particles: Properties and perspectives for bioapplications. Critical Reviews in Solid State and Materials Sciences.

[17] Perevedentseva E, Lin Y-C, Jani M, Cheng C-L (2013). Biomedical applications of nanodiamonds in imaging and therapy. Nanomedicine-Uk.

[18] Ho D, Wang CHK, Chow EKH (2015). Nanodiamonds: The intersection of nanotechnology, drug development, and personalized medicine. Science Advances.

[19] Dolmatov VY (2007). Detonation-synthesis nanodiamonds: synthesis, structure, properties and applications. Russ. Chem. Rev..

[20] Petit T (2012). Oxygen hole doping of nanodiamond. Nanoscale.

[21] Ginés L (2017). Positive zeta potential of nanodiamonds. Nanoscale.

[22] Zou Q, Li YG, Zou LH, Wang MZ (2009). Characterization of structures and surface states of the nanodiamond synthesized by detonation. Mater Charact.

[23] Krueger, A. *et al*. Deagglomeration and functionalisation of detonation diamond. *phys stat sol (a)* **204**, 2881–2887 (2007).

[24] Williams OA (2010). Size-dependent reactivity of diamond nanoparticles. ACS nano.

[25] Krüger A (2005). Unusually tight aggregation in detonation nanodiamond: Identification and disintegration. Carbon.

[26] Ozawa M (2007). Preparation and Behavior of Brownish, Clear Nanodiamond Colloids. Adv. Mater.

[27] Ushizawa K (2002). Covalent immobilization of DNA on diamond and its verification by diffuse reflectance infrared spectroscopy. Chemical Physics Letters.

[28] Krueger A, Lang D (2012). Functionality is Key: Recent Progress in the Surface Modification of Nanodiamond. Adv. Funct. Mater..

[29] Krüger A, Liang Y, Jarre G, Stegk J (2006). Surface functionalisation of detonation diamond suitable for biological applications. J. Mater. Chem..

[30] Shenderova OA, McGuire GE (2015). Science and engineering of nanodiamond particle surfaces for biological applications (Review). Biointerphases.

[31] Liang Y, Ozawa M, Krueger A (2009). A General Procedure to Functionalize Agglomerating Nanoparticles Demonstrated on Nanodiamond. ACS nano.

[32] Zhang X-Q (2009). Polymer-Functionalized Nanodiamond Platforms as Vehicles for Gene Delivery. ACS nano.

[33] Zhao L (2014). Polyglycerol-functionalized nanodiamond as a platform for gene delivery: Derivatization, characterization, and hybridization with DNA. Beilstein Journal of Organic Chemistry.

[34] Jana NR, Earhart C, Ying JY (2007). Synthesis of water-soluble and functionalized nanoparticles by silica coating. Chemistry of Materials.

[35] Hermanson, G. T. *Silane Coupling Agent*s. *BioconjugateTechniques* 535–548 (Elsevier, 2013). 10.1016/b978-0-12-382239-0.00013-3.

[36] Akiel RD (2016). Investigating Functional DNA Grafted on Nanodiamond Surface Using Site-Directed Spin Labeling and Electron Paramagnetic Resonance Spectroscopy. J. Phys. Chem. B.

[37] Bagwe RP, Hilliard LR, Tan W (2006). Surface Modification of Silica Nanoparticles to Reduce Aggregation and Nonspecific Binding. Langmuir.

[38] Brinker CJ (1988). Hydrolysis and condensation of silicates: effects on structure. Journal of Non-Crystalline Solids.

[39] Shenderova OA (2011). Nitrogen control in nanodiamond produced by detonation shock-wave-assisted synthesis. J. Phys. Chem. C.

[40] Volkov KV, Danilenko VV, Elin VI (1990). Synthesis of diamond from the carbon in the detonation products of explosives. Combust Explos Shock Waves.

[41] Girard HA (2010). Hydrogenation of nanodiamonds using MPCVD: A new route toward organic functionalization. Diamond and Related Materials.

[42] Cunningham G, Panich A, Shames A, Petrov I, Shenderova O (2008). Ozone-modified detonation nanodiamonds. Diamond and Related Materials.

[43] Shenderova O (2006). Modification of detonation nanodiamonds by heat treatment in air. Diamond and Related Materials.

[44] Abdulsattar MA (2013). Size variation of infrared vibrational spectra from molecules to hydrogenated diamond nanocrystals: a density functional theory study. Beilstein Journal of Nanotechnology.

[45] Wu Y, Wei P, Pengpumkiat S, Schumacher EA, Remcho VT (2016). A novel ratiometric fluorescent immunoassay for human α-fetoprotein based on carbon nanodot-doped silica nanoparticles and FITC. Anal. Methods.

[46] Singh G (2011). Detection of Bioconjugated Quantum Dots Passivated with Different Ligands for Bio-Applications. Journal of Nanoscience and Nanotechnology.

[47] Banyay M, Sarkar M, Gräslund A (2003). A library of IR bands of nucleic acids in solution. Biophysical Chemistry.

[48] Gaillard C (2014). Peptide nucleic acid–nanodiamonds: covalent and stable conjugates for DNA targeting. RSC Adv..

