# Supplementary material for: Functionalisation of Detonation Nanodiamond for Monodispersed, Soluble DNA-Nanodiamond Conjugates Using Mixed Silane Bead-Assisted Sonication Disintegration
Source: Sci Rep. 2018 Jan 15;8:728. doi: 10.1038/s41598-017-18601-6 (PMC5768878; doi:10.1038/s41598-017-18601-6)
Supplement: Supplementary file 1 — Supplementary Information [file 41598_2017_18601_MOESM1_ESM.pdf]

Supplementary Information:

Functionalisation of Detonation Nanodiamond for Monodispersed, Soluble DNA-Nanodiamond Conjugates Using Mixed Silane Bead-Assisted Sonication Disintegration

Robert Edgington[1,2,3*], Katelyn M. Spillane[1], George Papageorgiou[1], William Wray[1,4], Hitoshi Ishiwata[2], Marianna Labarca[2], Sergio Leal-Ortiz[2], Gordon Reid[1], Martin Webb[1], John Foord[3,†], Nicholas Melosh[2,†,*], Andreas T. Schaefer[1,4,†,*]

[1] The Francis Crick Institute, 1 Midland Rd, Kings Cross, London, NW1 1AT, UK
[2] Department of Materials Science and Engineering, Stanford University, Stanford, California 94305, United States
[3] Department of Chemistry, University of Oxford, Oxford, OX1 3TA, UK
[4] Department of Neuroscience, Physiology & Pharmacology, University College London, UK
[*] corresponding author r.j.edgington@gmail.com
[†] share senior authorship

Oligonucleotide Sequence

The following 3´ thiol-modified DNA nucleotide sequence (adapted from the Rothemund 2006 'sharp triangle' DNA origami design[1]) was used for DNA-DND conjugation:

(5´)TCTTTGATTAGTAATAGTCTGTCCATCACGCAAATTAAC
CGTTAAAAAAAAAAAAAAAAAAAAAAAAAAAAAAAAA(3´)–C3–SS

DNA-T80A20 aggregation resilience

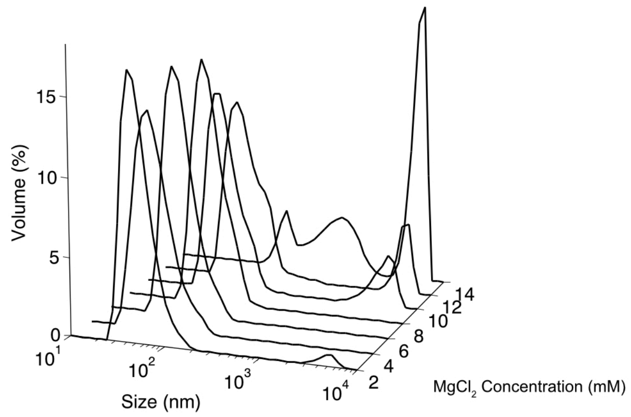

Figure S1: Size distribution of DND DNA conjugate vs. MgCl2 concentration. Aggregation is observed > 8 mM

Dynamic Light Scattering (DLS) size measurements were performed to determine the stability of DNA-T80A20 suspensions in solutions of varying divalent cation (Mg^{2+}) concentration (0-14 mM $MgCl_2$). Samples were mixed in 10 mM HEPES with 2-12 mM $MgCl_2$ and measured with DLS after a 5 minute stabilization period. The onset of aggregation is apparent at > 8 mM $MgCl_2$.

XPS Data

The following section shows the measured XPS spectra of DND–OH and TA samples used in this study, followed by a description of the XPS deconvolution algorithm used to estimate the amount of ATPES and THPMP present in each TA sample. C1s, O1s, N1s, Na1s, Si2p and P2p high resolution scans (as well as survey spectra) were measured for each sample and their integrated peak intensities were used for analysis as described below.

[1] Paul W K Rothemund, "Folding DNA to Create Nanoscale Shapes and Patterns" 440, no. 7082 (March 16, 2006): 297–302, doi:10.1038/nature04586.

Fig S2: XPS DND–OH

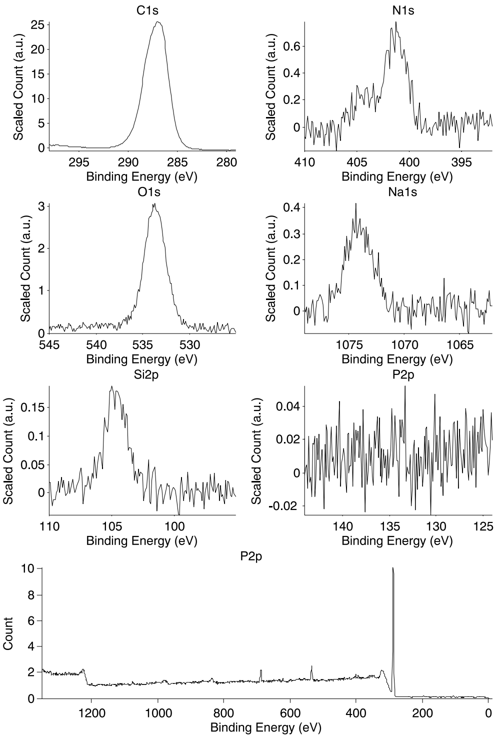

Fig S4: XPS T95A5

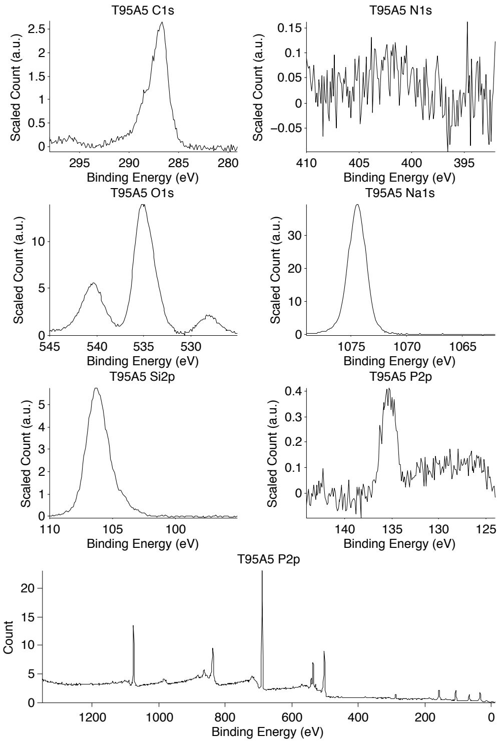

Fig S3: XPS T100

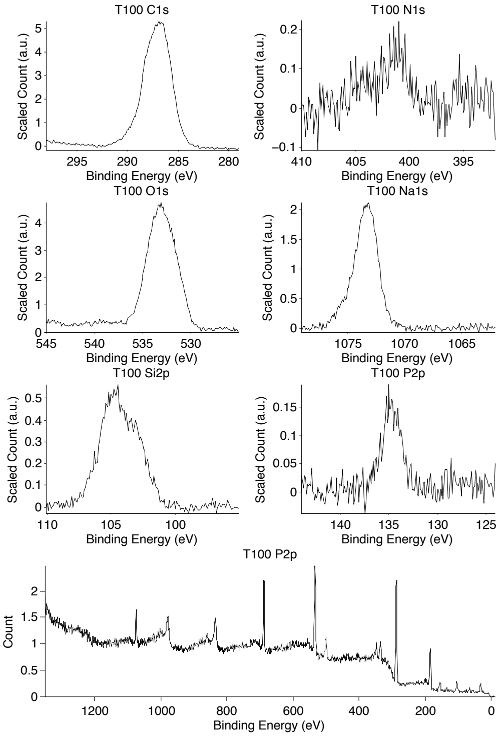

Fig S5: XPS T90A10

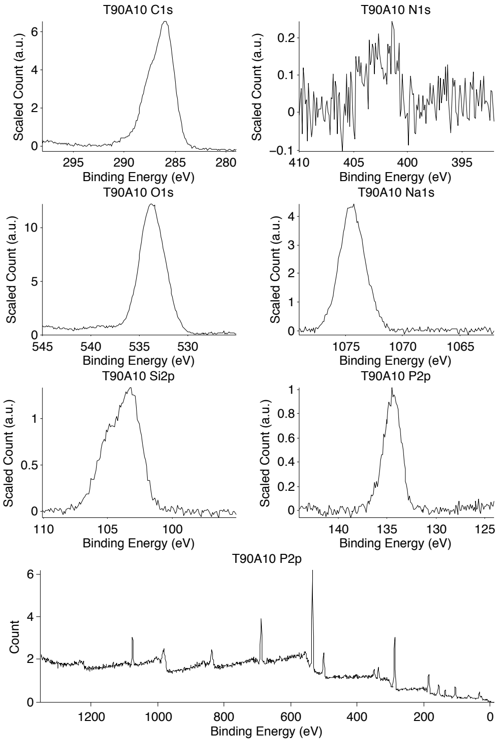

Fig S6: XPS T85A15

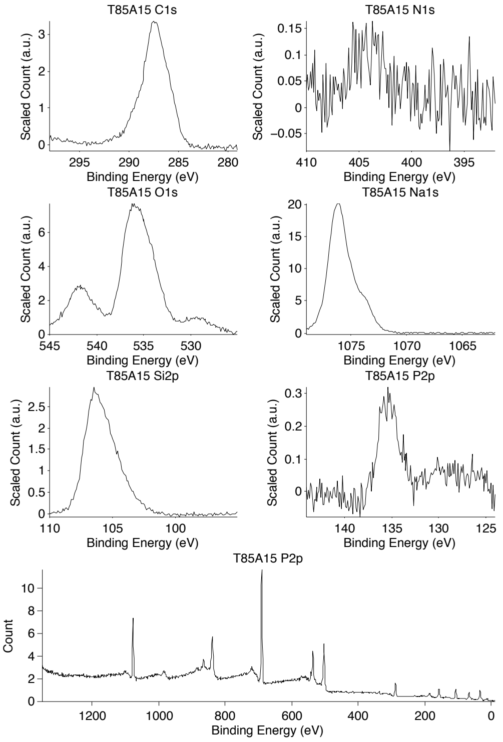

Fig S7: XPS T80A20

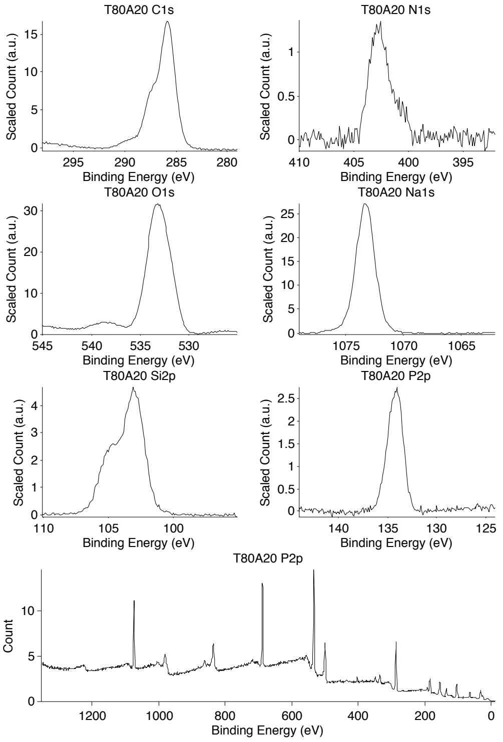

Fig S8: XPS T50A50

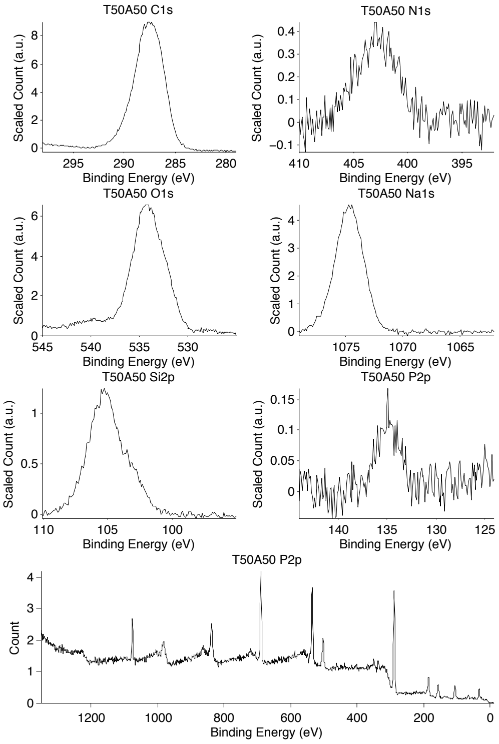

Fig S9: XPS A100

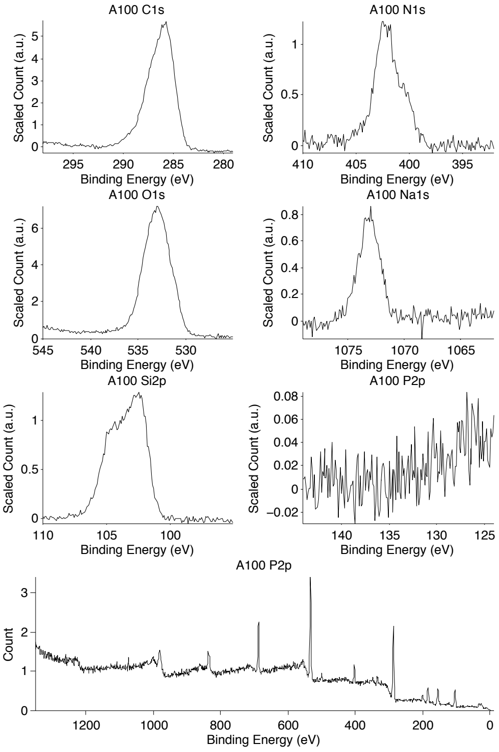

Percentage peak area (%)

	DNDOH	T100	T95A5	T90A10	T85A15	T80A20	T50A50	A100
C1s	77.34	58.86	10.40	40.89	20.78	34.47	59.09	47.17
N1s	1.22	0.98	0.30	0.73	0.42	1.56	1.61	5.86
O1s	4.50	27.70	36.54	38.85	34.33	37.34	24.09	32.40
Na1s	0.26	4.61	30.47	6.40	26.07	11.67	6.41	1.26
Si2p	0.51	6.88	21.32	9.71	17.34	11.62	8.35	13.22
P2p	0.04	0.97	0.97	3.43	1.07	3.33	0.46	0.09

Atomic Relative Sensitivity Factors (AMRSF)[2]:

C1s: 1
N1s: 1.66
O1s: 2.46
Na1s: 3.92
P2p: 0.442 + 0.865
Si2p: 0.308 + 0.604

For analysis and comparison between elemental high resolution scans for each sample, measured counts were normalized to account for variable elemental scan settings by division with the following equation for each peak:

Scan Periods * Pass Energy * AMRSF.

XPS scans were baseline corrected by subtraction of the lower-quartile of intensity over the elemental scan range.

N_{APTES} deconvolution from N_{XPS}

In order to estimate the relative amount of ATPES and THPMP in each TA sample, a linear model was constructed to deconvolve the relative contributions of APTES, THPMP and DND to integrated peak intensities across all TA samples. The model was derived from the known stoichiometry of silicon, nitrogen, and phosphorous in APTES and THPMP, and the relative percentage of intrinsic nitrogen in DND. This approach was used instead of conventional XPS peak deconvolution in order to avoid the subjective and erroneous task of deconvolving the highly overlapped and low signal-to-noise DND N1s and APTES spectra. The model was constructed as follows:

Assuming negligible contributions to the N1s signal from unaccounted-for sources, the integrated XPS N1s peak intensity (N_{XPS}) was defined as the XPS contribution of nitrogen from ATPES (N_{ATPES}) and the nitrogen intrinsic to the DND:

$$(1)\ N_{XPS} = N_{APTES} + C_{XPS} N\%_{DND}$$

Where $N\%_{DND}$ is the fractional percentage of nitrogen intrinsic to DND and C_{XPS} denotes the integrated XPS count of the carbon (or otherwise indicated element hereon).

---

[2] National Physical Laboratory, "Average Matrix Relative Sensitivity Factors (AMRSFs) for X-Ray Photoelectron Spectroscopy (XPS)," *AMRSF,* September 29, 2006.

Assuming all silicon contributions to originate from APTES and THPMP, and all phosphorous to originate from THPMP ($P_{XPS} = P_{THPMP}$), the total silicon was then expressed as the sum of N_{APTES} and P_{XPS}:

$$(2)\ Si_{XPS} = p_N N_{APTES} + p_P P_{XPS}$$

Where are real positive numbers $\{p_{N,P} \in \mathbb{R}_{>0}\}$ and introduce a scaling factor to account for discrepancies of measurement between attenuated signals in different chemical environments (e.g. escape depth), sensitivity factors, etc. Rearranging for N_{APTES}, equating the two expressions, and rearranging the right hand side to zero, the following expression was used to determine $N\%_{DND}$ and $p_{N,P}$.

$$(3)\ N_{XPS} - C_{XPS} N\%_{DND} - p_N^{-1} Si_{XPS} + \frac{p_P}{p_N} P_{XPS} = 0$$

Simplifying parameter notation.

$$(4)\ N_{XPS} - C_{XPS} N\%_{DND} - p_1 Si_{XPS} + p_2 P_{XPS} = 0$$

Due to low signal-to-noise ratio for some N1s measurements, fitting was improved (by obviating unphysical solutions) by inclusion of a regularization term to the objective function (4). This is equivalent to adding weights to the fitting procedure in MATLAB. The regularization term of a signal-to-noise estimate of the peak integration of the N1s scan (N_{RMS}) was used to de-weight noisy measurements.

$$(5)\ (N_{XPS} - C_{XPS} N\%_{DND} - p_1 Si_{XPS} + p_2 P_{XPS}) N_{RMS}^{-1} = 0$$

$N\%_{DND}$ and $p_{1,2}$ were then estimated by minimizing (6) using classical least squares fitting using the MATLAB lsqcurvefit() function and default trust-region-reflective algorithm for *i* TA samples where *i* = 1, 2, …, m (m = 7):

$$(6)\ arg\, min_{N\%_{DND}, p_{1,2}} \left(\sum_{i=1}^{m} \left(N_{XPS_i} - C_{XPS_i} N\%_{DND} - p_1 Si_{XPS_i} + p_2 P_{XPS_i} \right) N_{RMS_i}^{-1} \right)$$

Fitting the model to each TA sample XPS data, the following fit was obtained:

$N\%_{DND}$= 1.53% and $p_{1,2}$ = {0.36, 0.93} ($p_{N,P}$ = {2.82, 2.63}).

The fit $N\%_{DND}$ value of 1.53% was in close agreement to the T100 (0% APTES / N_{APTES}) measured value of 1.6%.

Finally, to obtain N_{APTES} estimates, the following formula was used:

$$(7)\ N_{APTES} = N_{XPS} - 0.0153 \times C_{XPS}$$
